# A qualitative analysis of information sharing for children with medical complexity within and across health care organizations

**DOI:** 10.1186/1472-6963-14-283

**Published:** 2014-06-30

**Authors:** Laura Quigley, Ashley Lacombe-Duncan, Sherri Adams, Charlotte Moore Hepburn, Eyal Cohen

**Affiliations:** 1 Family Medicine Centre, Toronto Western Hospital, 399 Bathurst Street, Toronto, ON M5T 2S8, Canada; 2 Division of Paediatric Medicine and Child Health Evaluative Sciences, The Hospital for Sick Children, 555 University Avenue, Toronto, ON M5G 1X8, Canada; 3 Lawrence S. Bloomberg Faculty of Nursing, University of Toronto, 155 College Street, Suite 130, Toronto, ON M5T 1P8, Canada; 4 Department of Paediatrics, University of Toronto, 1 King’s College Circle, Toronto, ON M5S 1A8, Canada; 5 Institute of Health Policy, Management and Evaluation, University of Toronto, 155 College Street, Suite 425, Toronto, ON M5T 3 M6, Canada; 6 CanChild Center for Childhood Disability Research, 1400 Main Street West, Room 408, Hamilton, ON L8S 1C7, Canada

**Keywords:** Information sharing, Communication, Paediatrics, Medical complexity, Health policy

## Abstract

**Background:**

Children with medical complexity (CMC) are characterized by substantial family-identified service needs, chronic and severe conditions, functional limitations, and high health care use. Information exchange is critically important in high quality care of complex patients at high risk for poor care coordination. Written care plans for CMC are an excellent test case for how well information sharing is currently occurring. The purpose of this study was to identify the barriers to and facilitators of information sharing for CMC across providers, care settings, and families.

**Methods:**

A qualitative study design with data analysis informed by a grounded theory approach was utilized. Two independent coders conducted secondary analysis of interviews with parents of CMC and health care professionals involved in the care of CMC, collected from two studies of healthcare service delivery for this population. Additional interviews were conducted with privacy officers of associated organizations to supplement these data. Emerging themes related to barriers and facilitators to information sharing were identified by the two coders and the research team, and a theory of facilitators and barriers to information exchange evolved.

**Results:**

Barriers to information sharing were related to one of three major themes; 1) the lack of an integrated, accessible, secure platform on which summative health care information is stored, 2) fragmentation of the current health system, and 3) the lack of consistent policies, standards, and organizational priorities across organizations for information sharing. Facilitators of information sharing were related to improving accessibility to a common document, expanding the use of technology, and improving upon a structured communication plan.

**Conclusions:**

Findings informed a model of how various barriers to information sharing interact to prevent optimal information sharing both within and across organizations and how the use of technology to improve communication and access to information can act as a solution.

## Background

Children with special health care needs (CSHCN) are those children who have or are at increased risk of a chronic physical, developmental, behavioral, or emotional condition and require health care and related services of a type or amount beyond that required by children generally [1,2]. Among CSHCN is a particularly vulnerable subgroup, known as children with medical complexity (CMC), who are characterized by substantial family-identified service needs, chronic and severe conditions, functional limitations, and extraordinarily high health care use [3]. While accounting for < 1% of all children, CMC consume almost one third of all paediatric health resources [4], and are at increased risk of multiple and prolonged hospitalizations, frequent medical errors [5], poor health outcomes [6], stress on family caregivers [7], and inefficient use of health care resources [8], which may be a result of poor care coordination [7].

The circle of care for CMC is extensive and includes many people (e.g. family caregivers, primary care providers, specialists, home care providers) that provide care in many places (e.g. the home, the school, camps, hospitals) over time. Consequently, the potential for children to experience gaps in their care is high. A comprehensive summative written care plan which is electronically accessible by all providers, as well as the patient, has been proposed as one effective method to achieve integrated and coordinated care. Health care providers (HCPs) and families collaborate to create a care plan which contains salient medical and psycho-social information, an updated list of medications, care providers, appointments, and goals of care [9]. The care plan is then integrated into the child’s electronic medical record, and can be updated during hospitalizations or clinic visits [9]. Various governing bodies [10-12] recommend that all CSHCN have an accessible, comprehensive, central record that contains all pertinent information about the child while maintaining confidentiality. Patients and caregivers acknowledge the importance of having access to this central record and sharing ownership of their health information with physicians [13].

Developing and implementing a written care plan requires effective information sharing (across providers, care settings, patients, and caregivers) to ensure appropriate and high quality care as well as perceived continuity of care by parents [14]. Suboptimal communication has been associated with unmet family needs, decreased family and physician satisfaction, unnecessary testing and treatment, and medical errors [15]. Given the large amount of health-related information that is generated during episodes of care across the continuum for CMC, a major challenge for care coordination is to consolidate current data (such as a written care plan) for these children into an easily accessible medium [16]. The use of electronic health information systems is a commonly proposed medium to improve communication between providers and patients/caregivers [17,18]. Advantages of an interoperable electronic system may include future financial benefits from avoiding redundancies and improving efficiency of administrative time [17,19] and from lower chronic disease management costs, lower medication costs, and lower wellness program costs [18]. Additionally, these systems can improve patient safety and quality of care [17] by reducing medical errors, improving continuity of care [18,19] and improving patient access to information to help them manage chronic disease [17,18]. The optimal medium would provide an ongoing connection between patient and physician, changing encounters from episodic to continuous [18].

While various jurisdictions have proposed models to create interoperability across electronic health information systems [20], studies have identified a number of difficulties introducing a common, secure platform including financial constraints (i.e. expensive, risky investment with uncertain returns) [21,22], limited interoperability between systems and other applications [17-19,21,22], privacy and security concerns [17,18,21,22], concerns regarding maintenance of the system [21], issues integrating the system across different organizations [18], the lack of a common language across disciplines [23], and the lack of a timely and easily accessible repository of medical information [16]. Concerns at the individual provider level have also been identified and include lack of time, heavy workload, motivation to use system, perceived ease of use, and familiarity with electronic systems [22].

There are not many examples of successful electronic care plan implementation [24]. Anecdotally it is known that there may be barriers to the sharing of written care plans for children with medical complexity [3]. The existing literature related to electronic system use and information sharing consists of mostly adult populations. Furthermore, the literature focuses on the sharing of a static document (i.e. an electronic health record). The sharing of a care plan requires families and health care providers across organizations to build and manage a dynamic document. Given the critical importance of communication through information sharing in high quality care of complex patients at high risk of poor care coordination, written care plans for CMC are an excellent test case for studying how well information sharing of a dynamic document is currently occurring. Understanding the barriers to information sharing for CMC may also be relevant to other high utilization, complex patients who require intensive medical services coordinated across multiple providers as well as a wide range of social supports, such as frail senior citizens [25]. The objective of this study was to understand the barriers and facilitators to the sharing of integrated paediatric complex care plans for CMC in Ontario, Canada from the perspective of parents, health care professionals, and privacy officers.

## Methods

### Study design

A qualitative study design with data analysis informed by a grounded theory approach was utilized. Grounded theory involves an iterative process whereby data is collected and analyzed cyclically to identify themes, until no new themes can be identified (theme saturation is reached). It is the optimal form of data analysis to generate a theoretical explanation of an understudied area [26]. Grounded theory was used to inform an overarching theory to the barriers to and facilitators of information exchange which has not yet been modelled in the existing literature for CMC. The study focused on identifying themes and subthemes from the data which were relevant to information exchange and considered either a barrier or facilitator. The RATS guidelines for reporting qualitative studies were adhered to.

### Setting

In Ontario, Canada’s largest province, health care services are funded by a single government payer (Ministry of Health and Long-Term Care (MOHLTC)), with funding allocated to 14 separate Local Health Integration Networks (LHINs). Each LHIN has an explicit mandate for planning, integrating, and funding health services at the local level [27]. Independent organizations (e.g. hospitals) and providers (e.g. physicians) primarily deliver health care in Ontario. Payments are allocated by LHINs to hospitals based on an accountability agreement developed between the LHIN and hospital, while providers are primarily paid through fee-for-service or an alternate payment plan which may include a mix of capitation, fee for service, special premiums and incentives, or salary by the MOHLTC.

This study was conducted at multiple organizations all geographically located within the Toronto Central LHIN, which serves approximately 1.5 million people [27]. Organizations included in this study were a 350 bed tertiary acute care academic hospital (The Hospital for Sick Children, SickKids), an organization who oversees care for patients at home and in the community in the Toronto Central LHIN, the Toronto-Central Community Care Access Centre (TC-CCAC), and a paediatric rehabilitation hospital who helps serve the developmental needs for many CMC (Holland Bloorview Kids Rehabilitation Hospital, HBKR). Most CMC in the Toronto-Central LHIN utilize services at all three organizations and together the three organizations provide the majority of care provision for CMC [4].

### Information sharing context

Health care providers and individuals or organizations receiving personal health information within Ontario are governed by the Personal Health Information Privacy Act [28]. PHIPA is based on implied consent within healthcare providers’ “circle of care” or on express consent from the individual. The purpose of PHIPA is to establish rules for the collection, use, and disclosure of personal health information that protects confidentiality and privacy, while facilitating effective provision of health care. PHIPA also requires health information custodians (a person or organization who has custody or control of personal health information) to respond to requests for access and correction, ensure transparency of information practices, protect the security of personal health information, and notify individuals if their personal health information is stolen, lost, or accessed by unauthorized persons [28]. PHIPA serves as a model for other health privacy statutes, and has been described by external jurisdictions as the “gold standard” among privacy statutes across Canada [29]. Furthermore, the United States Institute of Medicine recommended that its current health privacy statute (the Health Insurance Portability and Accountability Act) be amended based on PHIPA as a model [30]. Thus, many components of PHIPA are relevant across jurisdictions.

### Study population and data sources

The project assessed the facilitators of and barriers to sharing care plan information both within and between organizations by examining data from three different organizations (SickKids, HBKR, TC-CCAC), as well as primary care. Data was collected from 3 sources; 1) a study examining the desired content and perceived usefulness of care plans, 2) an evaluation of integration efforts across organizations for CMC, and 3) data from privacy officers from these organizations.

The first data source was secondary data from previously conducted semi-structured interviews with parents (n = 5) and focus groups with HCPs (n = 15) of CMC from a study focused on experiences with written care plans (Care Plan Study) [9]. All participants were recruited by a Research Assistant not involved in their clinical care. Parents of CMC participated in an in-depth, semi-structured interview at a time and location of their convenience lasting between 60 and 90 minutes. Individual interviews allowed for confidentiality to openly express thoughts. The interview guide questions explored parents’ experiences in creating and using the care plan, the meaning of having a care plan, and perceived key components and gaps in the care plan. HCPs participated in focus groups that were held at the Hospital for Sick Children and lasted 90 minutes. The focus group guide included questions exploring HCP understanding of care plans, their past use of care plans, perceived key components of the care plan, and perceived impact of care plans [9]. Although the sharing of care plan information was not the main focus of this study, many participants commented on the phenomenon.

The second data source was data from an evaluation study of a strategic initiative focused on integrated care for CMC in the TC-LHIN between SickKids, HBKR, and TC-CCAC (the Integrated Complex Care Model, or ICCM). This included interviews with parents of children with medical complexity (n = 12) and HCPs (n = 21), including those in manager roles, of CMC enrolled in the model. Participants were also recruited by a research assistant not involved in clinical care [31]. Among these providers were both those who created and maintained the care plans (nurse practitioners from the complex care program) and those that used the care plans (e.g. primary care providers). Interviews and focus groups were semi-structured and facilitated using a standardized guide. Individual interviews ranged from 20 to 70 minutes in length. Questions for key informants explored real-world application of the ICCM, as well as successes and lessons learned to support refinement, future replication, and expansion. Questions for parents explored family experiences with the model, perceived continued gaps in care, and overall satisfaction. Similarly, although the sharing of medical information was not the main focus of this study, many participants commented on the phenomenon [31].

To better understand information sharing from an organizational policy perspective, data was collected from a third data source, which consisted of semi-structured interviews with the privacy officers from SickKids, HBKR, and TC-CCAC. A privacy officer ensures all within their organizations is aware of their duties under PHIPA, and responds to and investigates any complaints about privacy practices [32]. Individual interviews with privacy officers lasted approximately 30 to 60 minutes and covered questions related to the barriers to and facilitators of the exchange of medical information for children with medical complexity that operate at the organizational level (e.g. procedures, policies, and priorities). Institutional ethics approval was obtained from all 3 partner organization ethics committees (The Hospital for Sick Children Research Ethics Board, the Bloorview Research Institute Research Ethics Board, and the Joint Research Ethics Board) and written consent was obtained from all participants.

### Data analysis

Grounded theory was used to inform an overarching theory describing the barriers to and facilitators of information sharing which has not yet been modelled in the existing literature. All semi-structured interviews and focus groups were audio-recorded, transcribed verbatim, and de-identified. Two independent coders (ALD and LQ) conducted secondary analysis of interviews with parents of CMC and health care professionals involved in care of CMC, and primary data analysis of interviews with privacy officers of associated organizations. Peer coding structures were compared to ensure trustworthiness. Open coding was used to form initial categories of the barriers and facilitators of information sharing, followed by a constant comparative approach with continual adjustment. Codes were examined, compared, merged, relabeled, and split as necessary. Upon completion of analysis, data saturation was obtained. Emerging themes related to the barriers and facilitators of information sharing were identified by the two coders and the full research team (LQ, ALD, SA, CMH, EC). Following a team meeting with the research group to review the open coding structure, an axial coding structure was developed and the data reassembled. NVivo 8 [QSR International, Australia] facilitated data management and coding.

## Results

### Participants

Data were obtained from a total of 56 participants. This included twenty participants from a study focused on experiences with written care plans, thirty three participants from an evaluation of integrated care in the TC-LHIN, and three interviews with privacy officers.

### Key themes

**Figure 1:**
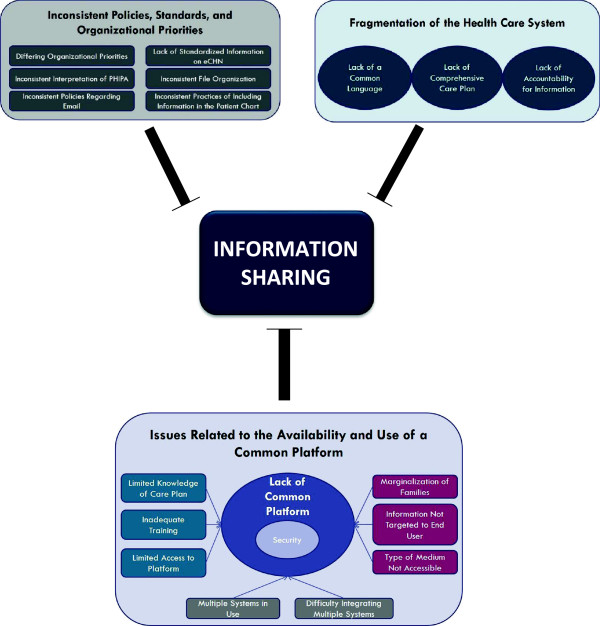
Barriers to information sharing.

Three major themes and various subthemes relevant to the barriers of sharing information, particularly written care plans, were identified (Figure 1); 1) issues related to the availability and use of a common platform on which a care plan is stored and accessed by all appropriate providers/families, 2) fragmentation of the current health care system, and 3) the impact current policies, standards, and organizational procedures have on the sharing of information.

### Barriers to information sharing

#### Theme 1: Issues related to the availability and use of a common platform

Families, HCPs, steering committee members (SCs), and privacy officers all identified various barriers related to the availability and use of a common platform for storing and sharing health related information. While many children may have an existing care plan, the location of this care plan varied both between services in the hospital as well amongst the various organizations.

#### Difficulty amalgamating multiple information systems and lack of a common platform in use across organizations

Currently, multiple information systems are in use across the various organizations which provide care for CMC, making communication difficult across sites as HCPs and families have to adapt to each system and the onus is on the individual to ensure they find all relevant up-to-date information. This theme was identified by HCPs, SCs, and privacy officers. Key stakeholders acknowledged that integration is particularly difficult in the absence of a good system level approach to data or patient information. This challenge was identified not only across the organizations but also within organizations.

*“Probably different systems [across the various organizations and/or] the fact that they are on different platforms [impacts on information sharing]. Whose system is it? I think because of the timing and because of our different perspective there will be times that information on one system will vary and there’ll be duplication but even contradictory statements.”* (Privacy Officer 2)

#### Difficulty accessing the care plan on a common platform

A key barrier to accessing information from a common platform is whether or not the provider has the knowledge that a care plan exists. If the family does not mention the existence of a care plan, or if it is not somehow flagged in the child’s file, the care plan may not be accessed. Oftentimes there was variable access to the appropriate platform on which the relevant information was stored across HCPs. While staff physicians may have access to the required platform, other providers within the circle of care (i.e. residents, fellows, community HCPs) may not, due to not having log in information or not having remote access, considerably impacting the efficiency of updating the child’s information. Finally, HCPs expressed concern that even if they had authorization to access a platform containing patient information, they may lack the appropriate training to do so.

*“…Staff Physicians have access to many of these documents on the electronic patient chart. The residents or the fellows do not have access. That alone makes this document probably useless to some extent”.* (Care Plan Study, HCP)

#### Concerns with the security of the platform

A key barrier related to the common platform itself, particularly the sharing of confidential information, is the lack of security. Many providers and privacy officers highlighted that even if a common medium for communication across organizations exists, such as the use of electronic mail, this medium was inappropriate for information sharing because it was perceived as not secure. The current use of a single encrypted email system across organizations (i.e. ONEMail developed by eHealth Ontario) does provide a secure tunnel across providers but has potential limitations (e.g., emails can be accidentally sent to the wrong recipient.)

*“An electronic Care Plan can be e-mailed to people who are participating in care. The problem with e-mail has obviously to do with confidentiality and security relating to different e-mail systems and servers.”* (Care Plan Study, HCP)

#### Concerns about how the platform is utilized to help share information within the circle of care

Family marginalization is a prominent concern in regards to relaying information within the circle of care. Many families felt that providers were communicating with each other but were not keeping the family updated and informed, especially by email. At the institution level, privacy officers acknowledged concern about how to include the family in information sharing, even within a common platform. Lastly, there is varying provider and/or family preference for the type of medium (electronic or paper) and the information to be included in the output from a common platform. Specific parts of a care plan may be more or less relevant to each provider and if the format and information included in a care plan is not tailored to each provider, the use of care plans and access to a common platform may remain limited.

*“And I have no idea how the other parts of the team are communicating. I’m not copied on anything over email. It’s a big black hole from the family perspective…..so that would be my first suggestion, is to copy me on [it].”* (ICCM Study, Family)

#### Theme 2: Fragmentation of the health care system

The next major theme identified was the current fragmentation of the health care system with a focus on episodes of care, rather than a continuum of care. This is particularly important for CMC as their care is ongoing and involves a number of different providers and settings. The lack of a focus on a continuum of care may be a contributing factor to the lack of clear expectations for ongoing information exchange as well as limited accountability for the health information.

#### Lack of common language across organizations and health care providers

A barrier to communication between organizations is that each organization uses a different language. For example, one organization may call a child a patient while another organization may call a child a client.

*“…we didn’t start even with a shared nomenclature or a shared vocabulary or a shared language about even how to review cases. Depending on which key worker started the conversation, you know, it was a very different approach.”* (ICCM Study, Steering Committee Member)

#### Lack of clear expectations for ongoing information exchange

It was clear that at many times a HCP was expected to piece different components of a patient’s care together to replicate the overall picture. The current system does not incorporate a comprehensive communication plan for the continuum of care.

*“I find when patients get admitted, we often find out when the discharge summary gets faxed to the office … And it’s a problem, because parents perceive that you’re involved, and you know what’s going on, and sometimes they don’t contact you and then their kid arrives in the office with a discharge summary, it’s not good. And it happens very often. Even from ICU admissions.”* (Care Plan Study, HCP)

#### Accountability for information

Both families and HCPs expressed concern over the lack of accountability for the care plan. While various expert panels recommend a comprehensive care plan for CMC, it is unclear who would oversee the care plan as a whole while each individual provider contributes their own piece of information. Accountability for the accuracy of the information is paramount to ensuring that patients receive high quality care.

*“Who’s going to consistently look at [the care plan] and consult with all the teams to make sure it’s followed. I think that’s kind of what I struggle with.”* (ICCM Study, Family)

*“You highlight one of the problems that sits in many institutions. Who is actually responsible and accountable for the overall care of the patient […] identifying the responsible or accountable owner of the care plan is paramount....”* (Care Plan Study, HCP)

#### Theme 3: Policies, standards, and organizational priorities

The third major theme of barriers to information sharing involves the policies, standards, and organizational priorities in place at the provincial level as well as the institutional level.

#### Inconsistent policies and standards across organizations regarding information sharing

The interviews with privacy officers illustrated that many of the requirements imposed by PHIPA are vague in their terminology. The quotes below show that “*unsecure*” and “*necessary*” are left to the interpretation of the organization. This has resulted in privacy officers creating varying standards and policies across the organizations. HCPs, managers, and privacy officers repeated numerous times the impact of inconsistent messages from each organization regarding the use of electronic mail. Individuals are unclear about whom they can contact with email, what information can be included, and how secure the email needs to be to ensure confidentiality and privacy. Interestingly, frontline HCPs were less conservative in their interpretation of PHIPA than SCs and privacy officers, especially in regards to the use of email.

*“…we have to interpret the Act, and the Act says you have to keep information private and not share it in an unsecure way.”* (Privacy Officer 1)

*“…That then goes back to the e-mail, whether or not it’s appropriate. It all depends on whom I’m contacting. Some [subspecialists] are very stickler about [communicating via email] because I have an outside account. I have an [organizational account] but I can’t access it from home and I’m only down here one day a week. So I just use my own e-mail and some of them will be fine, and some staff have said “I can’t talk to you”.* (Care Plan Study, HCP)

#### Organizational priorities

Currently the organizations develop internal policies or standards that focus on accountability for the care delivered within their own institution. Ostensibly this may mean that leaders at each organization are not focused on the overarching quality of care across the continuum for children who access care across settings.

*“All of us are in the business of looking after these kids, but our interface with each other is, even though it’s extensive, it’s not formally looked at and developed as strategic directions for any of our organizations extensively.”* (ICCM Study, Steering Committee Member)

#### Inconsistent format of care plans and file organization upon electronic systems

Users of a current centrally accessible electronic child health network (eCHN) speak to current limitations such as hospitals sharing varying degrees of information. Currently there appears to be a lack of standardized information, as well as a lack of standardized location, within the electronic system. Thus, locating the various pieces of information can be tedious and may complicate the communication process.

*“One of the things that has not occurred in the electronic patient chart here, although there are many sections that include different elements of the chart, there is not one for a Care Plan.”* (Care Plan Study, HCP)

#### Ensuring the patient information reaches the patient chart

Privacy officers voiced concern that when providers and families use a form of technology such as email to communicate about patient care, this information may not be added to the patient chart for other professionals to view. While one participating organization does have the capability to include email communication in the patient chart, it is unclear if practitioners always copy their email to the patient chart and whether other organizations have a similar system.

*“If we start doing our documentation in email it doesn’t get to the patient record. You don’t have the record that you did communicate. Although we do have a method to get emails to the record, most organizations don’t…….. And again that’s what I worry about if it’s easy to email and people communicate that way all the time, from a medico legal point of view you really want that to be part of the record.”* (Privacy Officer 3)

### Facilitators of information sharing

**Figure 2:**
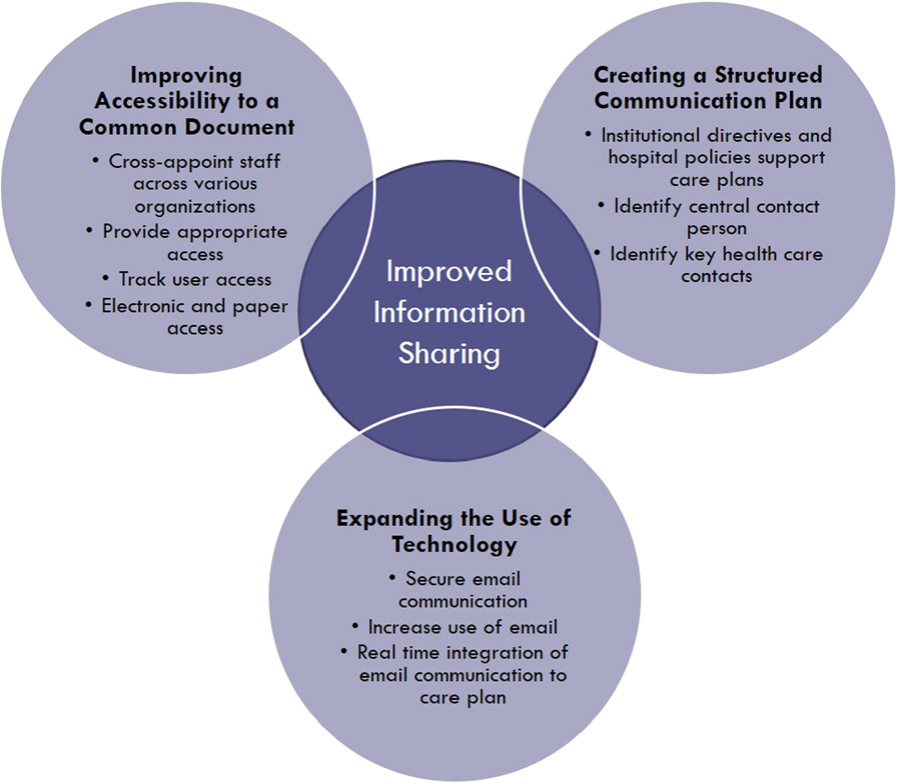
Facilitators of Information Sharing.

Key informants identified a number of facilitators to information sharing (Figure 2).

#### Theme 1: Improving accessibility to a common document

The first suggestion questions cross-organization privacy policies which prevent providers from viewing patient information from other organizations. Key informants recommended allowing all members within the circle of care to access relevant patient information and continue to provide family-centered care. One method of increasing fluidity across organizations is to cross-appoint staff at multiple organizations serving CMC.

*“…think having some model around staff being more fluid between the three organizations so that you are not just [one organization’s] employee but you somehow have a relationship with the other [organizations]…fluidity may be important”.* (Privacy Officer 3)

Discussion amongst families and HCPs about what information each HCP can be privy to may help to maintain some patient confidentiality across providers. A system which provides and tracks access to the patient information essential to each provider, may improve accessibility while maintaining confidentiality and security. While electronic access is important, a paper copy may continue to be necessary for families who do not have electronic access.

*“I think if more of the partners along the health care continuum and within the circle of care had access to a single integrated record and even if it only gave us access to parts or their access was tracked and individuals could say I’m okay with my GP having it or my pharmacist or whatever.”* (Privacy Officer 2)

#### Theme 2: Expanding the Use of technology

The second theme revolved around expanding the use of existing technology. The key characteristics reflected by families and HCPs included increasing the use of email and having real time communication. The limiting factor to expanding the use of technology appears to be finding a secure method. Privacy officers are open to expanding communication means however are still waiting for a secure method available across all organizations where emails cannot reach an unintended recipient.

*“So I think e-mail is huge…I've seen e-mail that has worked really well not only within but also across service providers and different agencies, so I think that's huge.”* (ICCM Study, HCP)

*“Our ultimate next step is to develop [the electronic system] so that we have that opportunity for real-time integration and data entry.”* (ICCM Study, Steering Committee Member)

#### Theme 3: Creating a structured communication plan

The third theme to improve information sharing for CMC was to create a structured communication plan. A structured communication plan starts with an institutional directive and hospital policies to promote the use of care plans. Key informants also highlighted the need to identify key contacts to ensure appropriate follow-up. Furthermore, a central contact person is essential for the family to oversee updates to the care plan. Key informants felt that the care plan can become confusing or incorrect if multiple people were providing updates.

*“Just speaking from a Nurse Practitioner standpoint, I think of my role as the glue that holds different pieces together…As long as there’s a consistent person [updating the care plan], then I think that it will minimize the risk of things being missed.”* (Care Plan Study, HCP)

## Discussion

Information sharing across providers and care settings is essential to providing seamless integrated care for CMC as they move between primary, acute, specialty, and community care [6,10,33]. Findings from our study imply that the interpretation of privacy regulations and comfort with using electronic means to communication became much stricter the further removed an individual was from patient care. Furthermore, the terminology within the privacy legislature is interpreted individually by the privacy officers at each organization, promoting discrepancies in policies developed across care settings. The remaining findings from varied participants and data sources are consistent with barriers to information sharing identified in the literature [10,14-18,21-23,34]. The barriers identified highlight the need for a common, accessible, secure platform upon which a care plan is stored and easily accessed and updated for CMC [33,34].

While the interaction between patient, caregivers, and healthcare providers impacts upon information sharing, the larger system and organizational structure are also important [23]. Studies reiterate the need for care that emphasizes coordination between sectors of the health care system, however, the health care system was largely designed to address acute episodes of illness rather than provide an ideal platform for addressing care for chronic conditions [14,35]. This may be exacerbated by fee-for-service funding structures that may promote a focus on episodic service delivery and communication [15], as opposed to system redesign that promotes chronic disease management by bundled payments, spread of registries and electronic information systems, and targeting the highest risk patients for outreach and follow-up [36]. The lack of a focus on the continuum of care within the current health system and inconsistent policies and standards provide further barriers to a central medium for information sharing [33].

Concerns regarding privacy and security are at the forefront of the discussion of expanding information systems to achieve a central medium for information sharing. While many patients support the idea of electronic medical records, a large majority are still concerned about the associated risks (i.e. identity theft, use of medical information for marketing purposes, employer access, insurer access) [37]. While the debate continues, privacy and security is not necessarily an obstacle to achieving advances and can help enhance public trust and confidence in the rapid adoption of electronic systems [37]. Cross-organization fluidity and increased use of technology, especially email, can increase access to a common document. Our study findings illustrate that there is a difference in priorities between those at the organizational level (i.e. privacy officers), and those on the front lines (i.e. health care providers and families). Privacy officers continue to express concern about the security of email (even through an encrypted server like ONEMail) and discourage its use in many situations. Health care providers are inconsistent in their use of email, likely because they are trying to adhere to the privacy laws passed along from their privacy officers meanwhile maintaining adequate communication with their patients. At the child and parent level, studies identified that parental concerns regarding privacy and confidentiality with the use of electronic means for communication are minimal [38]. In our study, families of CMC did not voice any privacy concerns. Parents were more concerned about inadequate communication, in many cases recommending more frequent use of email. This may be because parents of CMC are used to sharing information about their child with many different people involved in their child’s care to improve quality. Risk-benefit ratios need to be evaluated and some risks (such as emails being sent to unintended recipients) may need to be communicated to patients and their caregivers in the context of the overall goal of improved information sharing.

There are a few limitations to this study. Firstly, although we collected primary data from the privacy officers, the use of secondary data sources from informants participating in research that was only partially related to care plan information sharing means that the interview guides contained only a select number of useful questions. Given that this study consists largely of a secondary analysis of previously collected data, the identification of themes related to information sharing may have been less than if the interview guides focused solely on information sharing. However, given the ample amount of data available from the secondary data sources and triangulation through interviews with privacy officers, the study may be useful in helping to create an initial model of the barriers to and facilitators of information exchange. Although we collected data from many different types of informants across multiple organizations, the findings reflected the experiences and opinions of a limited number of English speaking participants in a single geographic region with a unique health care and legislative context which may limit transferability to other settings with different privacy legislation and/or health care information systems. Additionally, there are many key players located outside of the hospital or physician office setting (i.e. community based physiotherapists or occupational therapists) who were not interviewed for this study and would likely add an important perspective. However, the findings likely transcend across adult populations who are medically complex as they too require well-coordinated care and optimal information sharing to improve care [36]. The study focuses on CMC as an example of a medically complex population, thus the results may not be generalizable to other less complex populations, particularly those CSHCN populations who do not have as many providers and organizations involved in their care. Lastly, while the parents of CMC accepted the risks of e-mail communication, other groups of patients with ‘hidden’ but socially stigmatizing chronic conditions (e.g. mental health conditions) may be more risk averse.

## Conclusions

While a written care plan has been instituted for CMC within the structured complex care program at SickKids and within an integrated model of care, this study adds further insight to what may be limiting the sharing of these care plans and information. Families, providers, and privacy officers all recognize the multitude of barriers to information sharing for CMC and strive towards a better avenue of communication. As health care policy is now focused on improved efforts to integrate and coordinate care for complex patient populations, our findings suggest that careful thought into strategies that promote seamless communication of summative care plan information is an essential component of effective and efficient care for complex patient populations.

## Abbreviations

TC-CCAC: Toronto-Central Community Care Access Centre; LHIN: Local Health Integration Network; TC-LHIN: Toronto Central Local Health Integration Network; CMC: Children with Medical Complexity; CSHCN: Children with Special Healthcare Needs; HCP: Health Care Provider; ICCM: Integrated Complex Care Model; SC: Steering Committee Member; HBKR: Holland Bloorview Kids Rehabilitation Hospital.

## Competing interests

The authors declare that they have no competing interests.

## Authors’ contributions

LQ conceived of the study, participated in study design, participated in data collection, participated in data analysis and interpretation and drafted the manuscript; ALD participated in data analysis and interpretation and helped to draft the manuscript; SA participated in data interpretation and helped to draft the manuscript; CMH provided expertise in policy analysis, participated in data interpretation and helped to draft the manuscript; EC conceived of the study, participated in study design, participated in data interpretation and helped to draft the manuscript. All authors read and approved the final manuscript.

## Pre-publication history

The pre-publication history for this paper can be accessed here:

http://www.biomedcentral.com/1472-6963/14/283/prepub
